# Word embedding empowered topic recognition in news articles

**DOI:** 10.7717/peerj-cs.2300

**Published:** 2024-12-11

**Authors:** Sidrah Kaleem, Zakia Jalil, Muhammad Nasir, Moutaz Alazab

**Affiliations:** 1 Department of Computer Science, International Islamic University, Islamabad, Islamabad, Islamabad, Pakistan; 2 Department of Data Science & Artificial Intelligence, International Islamic University, Islamabad, Islamabad Capital Territory, Pakistan; 3 Department of Software Engineering, International Islamic University, Islamabad, Islamabad Capital Territory, Pakistan; 4 Department of Intelligent Systems, Faculty of Artificial Intelligence, Al-Balqa Applied University, Al-Salt, Jordan; 5 School of Computing and Data Sciences, Oryx Universal College with Liverpool John Moores University, Doha, Qatar

**Keywords:** Artificial intelligence, Computer vision, Neural networks, Natural language processing, Word embedding, Topic modeling

## Abstract

Advancements in technology have placed global news at our fingertips, anytime, anywhere, through social media and online news sources. Analyzing the extensive electronic text collections is urgently needed. According to the scholars, combining the topic and word embedding models could improve text representation and help with downstream tasks related to natural language processing. However, the field of news topic recognition lacks a standardized approach to integrating topic models and word embedding models. This presents an exciting opportunity for research, as existing algorithms tend to be overly complex and miss out on the potential benefits of fusion. To overcome limitations in news text topic recognition, this research suggests a new technique word embedding latent Dirichlet allocation that combines topic models and word embeddings for better news topic recognition. This framework seamlessly integrates probabilistic topic modeling using latent Dirichlet allocation with Gibbs sampling, semantic insights from Word2Vec embeddings, and syntactic relationships to extract comprehensive text representations. Popular classifiers leverage these representations to perform automatic and precise news topic identification. Consequently, our framework seamlessly integrates document-topic relationships and contextual information, enabling superior performance, enhanced expressiveness, and efficient dimensionality reduction. Our word embedding method significantly outperforms existing approaches, reaching 88% and 97% accuracy on 20NewsGroup and BBC News in news topic recognition.

## Introduction

Information is now abundant on the internet, and social media has changed how we access and use it. The rise of social media has transformed, with tons of information and news now available online (Ahmad et al., 2023). Online media platforms from blogs and social media to dedicated news websites offer an unprecedented variety of information in the form of text, video and images. With fast updates on mobile devices, the internet has expanded the variety and accessibility of news. This has produced a flood of information, making it difficult to locate relevant information.

Extensive electronic document collection demands media classification techniques. User-tailored content has a direct effect on their happiness. Consequently, content categorization has emerged as a vital and popular technique in non-news domains such as sentiment analysis, content retrieval, and spam filtering.

Topic modeling is a method of unsupervised learning that aims to identify hidden topic patterns in a collection of text documents. The main advantage of unsupervised methods is that they do not require predefined data for training. However, this variability comes with a trade-off: topic models may have lower accuracy and require longer training times compared to supervised methods (Du et al., 2022). A popular topic model of probability is latent Dirichlet allocation (LDA). LDA documents assume a mixture of hidden topics, each word in the document is based on the probability of being associated with those topics. This allows LDA to automatically identify topics from larger documents using possible terms will be distributed across different areas (Rashid et al., 2023).

LDA essentially transforms the original document-term matrix into a low-level hidden semantic space (Du et al., 2022). This reinforced representation captures the thematic relationship between words. The model assumes that documents are created by first selecting a range of topics (*e.g*., politics, sports) and then selecting words related to those topics. Topic modeling techniques such as LDA then analyze the co-occurring terms to retrieve these hidden thematic structures in the data *i.e*., find out that “soccer” and “ball” come from the same topic (Mikolov et al., 2013).

Hidden words capture meaning and relationships between words by assigning complex vectors of numbers to higher space. These vectors provide semantic information and facilitate comparisons between words based on similarities in meaning and context (Roman et al., 2021). One limitation of traditional methods such as “bag of words” is their reliance on word frequency alone, which tends to be low when used in a variety of contexts These methods lack the ability to capture meaning in small details based on the surrounding words. Advanced word representation techniques, including word placement, have overcome this limitation by incorporating contextual information into word representations (Elhassan et al., 2023). Because of its ability to capture the semantics (meaning) of words and the nature of a particular task, word placement has proven to be effective in a variety of industries (Liu et al., 2018). As a result, the performance of the models associated with the textual data was significantly improved. One of the most commonly used sets of terms is a search for similar terms. For example, using cosine similarity, a model might return “Spain,” “Belgium,” “Netherlands,” and “Italy” as words similar to “France.” Interestingly, word deposition vectors also exhibit statistical properties, such as linear relationships between words with similar meanings (Mikolov et al., 2013).

Furthermore, topic structure and word placement capture valuable aspects of meaning in a text by focusing on specific instances, each providing a partial perspective. This opens exciting opportunities for integration and comprehensive writing representation. However, the integration of content and lexical representations is a particular challenge in text analysis, where a single established methodology does not exist (Jalil, Nasir & Nasir, 2021). Existing models, most commonly using word2vec, may miss subtle nuances in word meaning due to their reliance on single, context-free embeddings (El-Affendi, Alrajhi & Hussain, 2021).

This complexity arises from several factors, including:

- The unique demands of different tasks (Nasir, Ikram & Jalil, 2022).
- The constant evolution of text representation models (Narayan, Cohen & Lapata, 2018).

Current research explores methods to enhance the topic identification accuracy of news data, but limitations persist. These limitations often involve trade-offs, such as increased model complexity and training time, while balancing the granularities of topic information represented by word or document distributions. In light of these challenges, we propose a novel topic model that integrates word embeddings for large text documents. This fusion approach aims to tackle the aforementioned limitations by unlocking structural information within both topics and words, resulting in the generation of high-fidelity topics, capturing a richer and more nuanced understanding of the text data.

We develop a full-process topic recognition system for news content and suggest a novel approach to text representation in response to the above limitations. This article proposes the newly developed approach which is based on resampling words using information from the word embedding models. Our model, called WELDA (for word embedding LDA), that leverages information from word embeddings along with the modified Gibbs sampling algorithm. We further analyze the different topic models and word embedding enhanced topic models in the standard tasks of document classification for topic models.

We provide the following significant contributions:

- We present a new topic model called WELDA. In the embedding space, WELDA learns a multivariate topic distribution for each topic. This distribution is then used to sample new, similar words for existing topics, thus, improving the topics. We will present the full inference algorithm, discuss the implementation details and thoroughly evaluate WELDA.
- The framework based on news text data is presented. The preprocessing layer, feature extraction layer, and topic recognition layer are the three core modules that make up the framework. The required data cleaning is handled by the preprocessing layer, and the suggested text representation technique is implemented in practice using various vector concatenation techniques by the feature extraction layer. Additionally, random forest and logistic regression (LR), two well-liked classifiers, are used in the topic recognition layer. The whole architecture makes use of technologies like word embedding, machine learning, BOW, LDA, and so forth.
- Several comparable techniques were chosen to serve as the baselines for this article. We carried out a thorough analysis using the two benchmark datasets from 20NewsGroup and BBC News.

## Related work

Several methods were proposed to model natural language reading. El Zein & da Costa Pereira (2022) used document collection to improve topic modeling by capturing syntactic and semantic regularities of words. A novel mathematics curriculum combining document collection and topic sampling was proposed. A collapsed Gibbs sampling algorithm was used and focused on quantitative and qualitative analysis. Stein, Jaques & Valiati (2019) worked on a comparative study to improve the hierarchical text classification (HTC) method for automatic document classification. The study observed that how traditional classification metrics differed from hierarchical classification metrics.

Furthermore, an investigation was conducted on the effects of various transcript representation methods and classification schemes on HTC. To evaluate the effectiveness of the models, researchers used a variety of evaluation criteria, including recall, precision, accuracy, F1 measurement, and specific design requirements.

Dieng, Ruiz & Blei (2019) developed the generative document model that checks which works better in terms of topic quality and prediction performance by integrating dynamic latent Dirichlet allocation (D-LDA) with word embeddings. Two D-LDA transformations were compared to D-ETM: the original (Blei & Lafferty, 2006) model and technique, and the D-LDA-REP model, which was fitted using mean-area variation estimates using the rescaling approach.

Furthermore, Bianchi, Terragni & Hovy (2021) improved the topic. In order to improve the coherence of the selected subjects, the challenge was resolved by mixing neural themed samples and contextual texts from BERT. The results showed that their pattern set technique is the second-strongest model in terms of performance, especially when applied to the 20NewsGroups dataset.

Three different metrics were used to evaluate the model: normalized pointwise mutual information (τ) and topic coherence (α) for external vocabulary reserves, and a metric to measure topic solutions.

Harandizadeh, Priniski & Morstatter (2022) offered a new approach called KeyE™ that introduced keyword priors to effective subject matter modeling algorithms. In addition, KeyE™ was appreciably examined on diverse today’s baselines to demonstrate its super overall performance in quantitative and qualitative facts assessment. Seifollahi, Piccardi & Jolfaei (2021) used enter phrases as classification factors. Their proposed set of rules worked on elements: first, it used a clustering approach with n-gram-word embedding to determine the topic word distribution, through a sampling process and matrix factorization, the document-topic distributions are generated. The cautioned model achieved an accuracy score of up to 25.71% above the previous one, outperforming all baselines in all information sets, in line with the outcomes.

The research of Rashid et al. (2023) improved topic coherence in short text by combining word input with topic mapping. To select relevant topics and gain understanding around them, a word entry format topic model (WETM) was constructed. Additionally, a modified collapsed Gibbs sampling strategy was proposed to increase the coherence of subjects in consensus briefs. In terms of classification results, the proposed topic model performed better than the original model.

To solve the problems of readability and sparsity, Gao et al. (2019) proposed a new model called sense unit-based phrase topic model (SenU-PTM). SenU-PTM on document charachterization and topical qualilty on search results based on two publicly available real-world datasets.

Other comparable systems that were already in use were more complex and failed to take into account the merging of word embeddings of data with topic modeling. To solve this issue (Du et al., 2022) developed a comprehensive framework for media topic detection in response to the shortcomings of existing frameworks in visualizing the integration of word input data and topic segmentation at different granularities. The collection improvement was made possible by a new word-based translation technique. It was shown that by combining the topic model and the entry word model, syntactic relations, semantic knowledge, and topic distribution can be effectively used to obtain better text representation.

In the research article of Gao et al. (2019), the LDA problem of sparsity was also briefly addressed. Another metric was used to determine distance and bundled bits of information into fake documents. Nonnegative matrix factorization (NMF) based topic modeling methods are popular for extracting salient features and discovering latent structures in text corpora. However, traditional NMF topic modeling suffers from high computational complexity and the risk of getting stuck in bad local minima (Zhang et al., 2019).

Table 1 summarized the key findings of the traditional methods used in topic modeling. While traditional models have offered valuable insights in the field of NLP, they have inherent limitations, the dataset used in the experiments is limited, which impacts the overall reliability of the results. The accuracy of the model also needs improvement. The topic-word and document-topic matrices, as well as the model itself, have only been computed once, which may limit robustness. Furthermore, the model has high time complexity and performs marginally slower than LDA across various topic counts. It struggles to represent social issues and less dominant topics effectively. This article investigates these limitations and proposes that WELDA can provide a more comprehensive understanding of topic modeling and word embeddings.

**Table 1:** A summary of the related work.

References	Datasets	Objective	Results	Limitations
Du et al. (2022)	20NewsGroup, BBC News	To improve topic recognition accuracy and effectively utilize key feature information such as syntax and semantics in the text	Micro-F1 scores = 0.810, 0.799	1. The Technique used in this article is simple, Deeper integration concepts have not been taken into consideration.
				2. Data used in the experiment is limited
Rashid et al. (2023)	Web Snippet and Amazon Review	To Improve topic coherency using word embedding in short text	Accuracy = 88%, 86%	1. Accuracy needs to be improved.
			Topic Coherency of web snippet dataset = −826.03	2. Model worked for only short text documents, although they apply collapsed Gibbs sampling algorithm which originally used for larger text
			Topic Coherency of amazon review dataset = −855.52	
Seifollahi, Piccardi & Jolfaei (2021)	Australia’s Victorian Government’s accident compensation agency	To leverages word co-occurrence and embeddings in topic modeling	Accuracy: D1= 66.78 %, D2 = 78.24%, D3 = 80.72	1. The topic-word and document-topic matrices have only ever been computed once, and the model has only been computed once.
Demeniconi & Chawla (2020)	Twenty-Newsgroup Dataset, Reuters-21578	To carry out document clustering and topic modelling concurrently in order to capture the syntactic and semantic regularities of words.	Clustering assessment in terms of accuracy = 71.89%, 45.84%. Topic modeling performance = −153.2415, −198,1154	1. Due to the complexity of the proposed methodology, It is impossible to derive the posterior distribution under EXPLORE.
Gao et al. (2019)	English news articles dataset. Stack Overflow dataset	To solve the sparsity problem of LDA in short text	Accuracy = 77.40%, 77.03%	1. The proposed model has high time complexity
				2. When comparing the suggested model to LDA across various topic counts, it performs marginally slower.
Harandizadeh, Priniski & Morstatter (2022)	AYLIEN COVID-19 dataset 20Newsgroups *corpus*,	To improve topic modeling by leveraging informative topic-level priors over the vocabulary	AYLIEN dataset: Precision = 0.61 Recall = 0.58 F1 (Marco) = 0.57 F1 (Micro) = 0.63	1. The model fails to represent the social issues topic.
			20newsgroup dataset: Precision = 0.84 Recall = 0.83 F1 (Marco) = 0.83 F1 (Micro) = 0.83	2. Unsuccessful in the less dominant topic
Bianchi, Terragni & Hovy (2021)	20Newsgroups Wiki20K, Stack Overflow Tweets2011 Google News	To improve the quality of the discovered topics	Average Topic Coherency: Wiki20K = 0.18 SO = 0.03 GN = 0.12 Tweets 20 = 0.10 NG = 0.10	1. A document’s remaining content will be lost if it exceeds SBERT’s sentence-length restriction.
				2. Fixed size embedding
Lu, Zhang & Du (2021)	Online snippets	To address the sparsity and readability Problems	Topic Coherency = −624.31, −1,064.84	1. The model does not tackle the word sense disambiguation (WSD) problem
			Accuracy: Online snippets = 83.77%	2. Accuracy needs to be improved.
			Online questions = 48.98%	
Nassif & Fahkr (2020)	20NewsGroup dataset	To captures both the semantic and syntactic meaning in global & local structure of words sequence	Produced a 1.4% to 2.4% improvement in performance over the to the state-of-the-art models	1. Higher computational time.
				2. Employed a supervised methodology in order to rely on labelled data
Dieng, Ruiz & Blei (2019)	UN Dataset = SCIENCE articles, ACL Antholog	To improve topic quality	UN: TC = 0.1206, TD = 0.6703, TQ = 0.0809	1. The Model requires structured variational inference with a recurrent neural network to fit the model, which may add complexity to the implementation process
			SCIENCE dataset: TC = 0.2298, TD = 0.8215, TQ = 0.1888, ACL dataset: TC = 0.1630, TD = 0.8286, TQ = 0.1351	2. It requires some computational resources and may not be practical for very large datasets

Table 2 describe some widely used topic modeling methods as the existing methods such as Latent Dirichlet Allocation (LDA), Latent Semantic Analysis (LSA) and Biterm Topic Model (BTM) with our approach WELDA for comparison.

**Table 2:** Comparison of traditional methods with WELDA.

Feature	LDA	LSA	BTM	WELDA
Topic coherence	Low	Medium	Medium	High
Handling polysemy/synonymy	Poor	Medium	Medium	Excellent
Computational complexity	High	Medium	Medium	High
Performance on large dataset	Poor	Medium	Medium	Excellent

The model LDA often produces topics that are not highly coherent due to treating words independently also it treats each word as a separate entity. LDA has high complexity due to iterative processes and struggles with scalability and efficiency on large datasets (Liu et al., 2023). While LSA performs better than LDA in terms of coherence because it captures some semantic structure. The LSA has medium computational complexity due to matrix decomposition and moderate performance on large scale datasets due to dimensionality reduction (Manalu, Arif Bijaksana & Suryani, 2019). The BTM has better coherence than LDA in short texts but still limited, it performs moderately well on large data, but scalability can be an issue (Huang et al., 2020). Whereas WELDA provide high topic coherence due to the integration of word embeddings, which capture semantic relationships between words. Low complexity in comparison because word embeddings pre-trained on large corpora reduce the need for extensive iterations. It provides excellent performance on large datasets due to the efficiency and scalability of pre-trained word embeddings.

### The proposed technique

**Figure 1:**
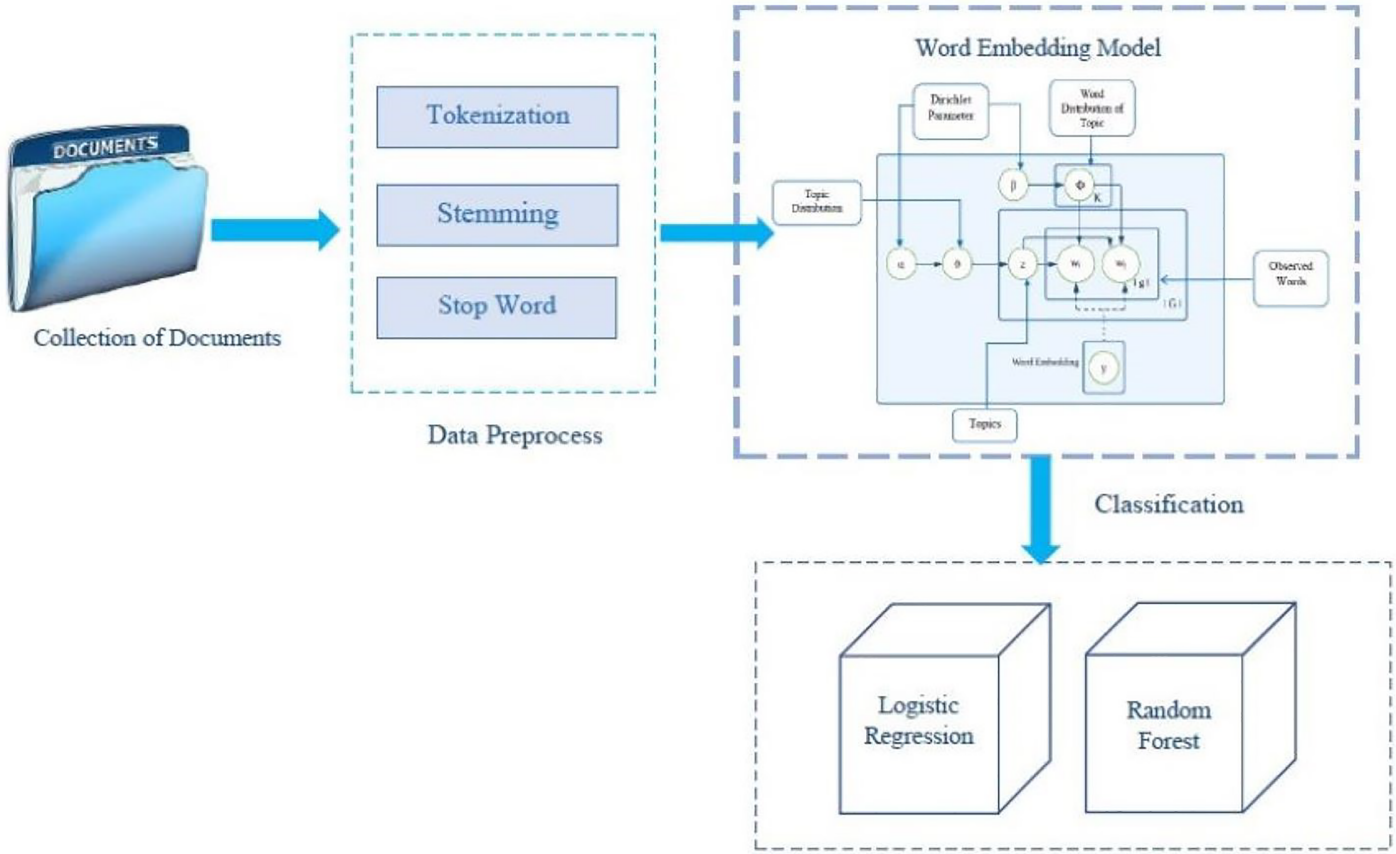
The architecture scheme of our proposed model.

The proposed approach consists of three main parts: preprocessing, classification, and feature extraction. Following is the discussion of the motivation behind our suggested method:

Figure 1 shows the architecture scheme of our proposed model. This architecture consists of key components like, pre-processing layer, including the embedding layer combined with topic modeling layer, and the classification module.

### Motivation

The motivation behind this proposed model is to devise a framework for combining the two methods, topic modeling and word embedding. Word embedding-based prior techniques prohibited topics that represent global word correlation. Words are only placed into the same topics if their underlying vectors have a strong geometric similarity, which produces low quality (Rashid et al., 2023). There is no standard framework for fusing word embedding representation with topic information specifically for large text collection (Du et al., 2022). In this research article, our goal is to investigate any possible overlaps between these technologies.

### Pre-processing

Pre-processing is a necessary step in natural language processing (NLP) projects which converts unstructured, raw data into a neat, structured format that allows algorithms to analyze it efficiently.

We will implement the following preprocessing functions on the *corpus*. The data-preprocessing phase includes tokenization, stemming, and stop word removal. Stop words are words like “a”, “the”, or “in” which don’t convey significant meaning, we removed those words that occur commonly across all the documents in the *corpus*. Next, we will leverage a stemming algorithm to transform words into their root forms, facilitating further linguistic analysis and information retrieval. Afterward we implement the tokenization function by breaking the raw text into words, and sentences that we can call tokens.

### Description of proposed topic model

The symbolizations used in the article are displayed in Table 3.

**Table 3:** Notations.

Variable	Description
D	Number of documents
W	Number of words
|g|	Different words
Y	Word embedding
|V|	Vocabulary size
G	List of related terms

The Table 3 provides a clear reference for understanding the various symbols and abbreviations used in the analysis.

This section contains the details of our proposed method, showcasing how we combine word embedding and LDA through a modified collapsed Gibbs sampling algorithm for optimal topic discovery.

**Figure 2:**
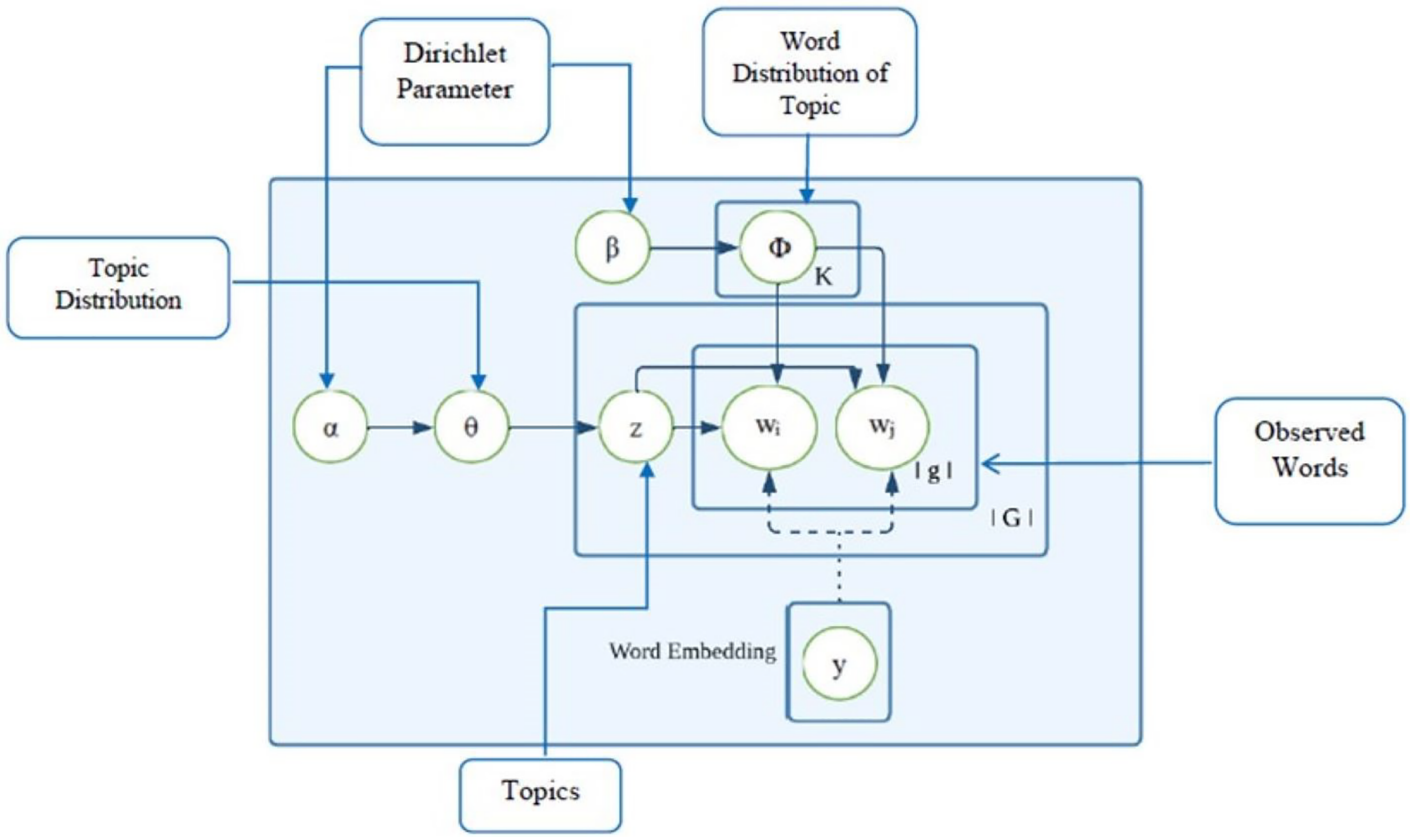
Word embedding latent Dirichlet allocation (WELDA).

The Fig. 2 illustrates the suggested topic model WELDA graphically. The suggested topic model preserves contextual information while maintaining each word’s semantic meaning.

Our suggested approach is used to refine topic models; we integrate the two approaches. We began by investigating how the outcomes of one model may be understood in the other since word embeddings and LDA topic modelling are unrelated methodologies with distinct origins.

We aim to combine the two methods, the LDA topic model and word2vec, a word embedding model. LDA topic model lacks semantic while the embedding model are good at capture semantic relationships between words. The word embedding models are trained on predicting a certain word at an exact position. In other words, if we look at the words that are similar to a given term, the model predicts a word that might have been in the same place. Based on the LDA topic model, this study suggests adding word2vec embedded model as an auxiliary information.

The goal is to transfer probability mass in the topic model more towards these words, which could have occurred at the same position. To do this, we take new word samples from the embedding space. We want to find hidden topics within the text by replacing words with others that fit better in their respective areas. To do this, we create a “topic distribution” for each area. This distribution tells us how likely different words are to appear in that area. We build these distributions by looking at the most frequent words in each topic and their location on the embedding space. Now, we can replace words in the text with others from their respective topic distributions. We use a special technique called “Gibbs sampling” to do this. This way, we gradually shift words towards their “natural” areas, revealing the hidden topics within the text. by sampling a new word in the Gibbs sampling algorithm’s innermost loop and then acting as though the newly sampled word had already appeared. These terms are quite similar to the most popular terms in the subject. Algorithm 1 Simplifies the inference process for the proposed topic model.

**Algorithm 1:** Proposed model WELDA with Gibbs sampling.

**Input:**
Documents D: A collection of text documents represented as lists of words. Topics Z: An initial assignment of topic labels to each word in the documents. Hyperparameters α, β
**Process:**
WELDA()
Create a vocabulary of unique words from the training *corpus*.
Assign each word a unique integer ID.
Initialize two matrices with random values:
Input word matrix (W): Size V × N (V = vocabulary size, N = embedding dimension)
Output word matrix (W′): Size N × V (transpose of W)
Set hyperparameters: embedding dimension, window size, learning rate, and negative sampling rate.
Train the model to predict a low probability for the negative word given the target word.
Estimate Distribution Parameter ()
**for** topic id $\in$ {1 … K} do
top words $\leftarrow$ Get top Ntop words in topic topic_id
topic vectors $\leftarrow$ [embedding matrix[word] for word in top words]
topic distributions [topic id] $\leftarrow$ estimate distribution (top vectors)
** for** d_idx $\in$ {1 … |D|} do
doc $\leftarrow$ D[d_idx]
** for** w_idx $\in$ {1 … |doc|} do
w $\leftarrow$ doc [w_idx]
t $\leftarrow$ Z [d_idx] [w_idx]
if true
sample $\leftarrow$ sample from distribution(t)
update w, as if we had seen another word
w = nn search.Find nearest word(sample)
decrement Z counters
t $\leftarrow$ sample new topic(Z)
increment Z counters
** End for **
** End for**
**End for**

For our proposed algorithm, Initially, first start with an initialization step which is run the word embedding model. Load a word embedding model. For all words in the *corpus*, we then retrieve its high-dimensional representation in the embedding space. Finally, using the lower-dimensional new word vectors, we initialize a data structure, that allows us to quickly find the nearest word given a vector in the embedding space. The suggested algorithm estimates the topic distributions for all subjects prior to each iteration of Gibbs sampling. Later in the sample procedure, we make use of these topic distributions in the innermost loop. Afterwards, instead of updating the matrices based on the actual word observed, we simulate the scenario where the sampled word appeared and adjust the document-topic and topic-word counts accordingly. The implementation of this model is on GitHub (https://github.com/SidrahKaleem/Topic-Modeling-with-Embedding).

### Intuitive justification for using Gibbs sampling algorithm

The basic intention behind using this algorithm among all the algorithms present in literature is that Gibbs sampling algorithm is a well-established approach for topic modeling, known for its efficiency in handling large datasets and its ability to parallelize for faster computation. Modifying it can potentially address limitations like slow convergence or poor performance on specific types of data. By modifying Gibbs sampling, we can tailor the algorithm to better suit the specific needs of WELDA.

### Experiment and evaluation

#### Dataset

Two widely used news text datasets, BBC News (https://www.kaggle.com/datasets/ervishvanathmetkari/bbc-news) and 20NewsGroup (https://scikit-learn.org/0.19/datasets/twenty_newsgroups.html) are chosen for this work based on the task criteria. The topic category tags on this text data indicate that they are all real-world sources.

Table 4 gives overall description of the 20-newsgroup dataset and BBC news dataset. This table helps in understanding the scope and scale of each dataset used in the study.

**Table 4:** The information of the datasets.

Feature	20Newsgroups	BBC news
Number of documents	20,000	2,225
Number of words	2 million	5.5 million
Vocabulary size	11,350	26,000
Average words per documents	100	2,500
Number of classes	20	5

#### Evaluation metric

The proposed approach is measured based on performance parameters.

#### Topic recognition

The topic recognition in news texts consider as a classification issue for the large text. In topic modeling, topic recognition refers to the ability to identify the most relevant topic(s) for a specific document. The suggested model for the single label multi-classification problem is evaluated using the Micro-F1 score.

$Micro \!-\! Precision = \; \displaystyle{{\mathop \sum \nolimits_{i = 1}^n T{P_i}} \over {\mathop \sum \nolimits_{i = 1}^n T{P_i} + \; \mathop \sum \nolimits_{i = 1}^n F{P_i}\; }}$

$Micro \!-\! Recall = \; \displaystyle{{\mathop \sum \nolimits_{i = 1}^n T{P_i}} \over {\mathop \sum \nolimits_{i = 1}^n T{P_i} + \; \mathop \sum \nolimits_{i = 1}^n F{N_i}\; }}$

$Micro \!-\! F1 = \; \displaystyle{{2\; \times \; Micro \!-\!  Precision\; \times \; Micro \!-\!  Recall\; } \over {Micro \!-\!  Precision\; + \; Micro \!-\!  Recall}}.$

#### Classification

We perform tests using topic distributions for classification. The evaluation of topic models includes an external task of text classification. Each document’s topics are regarded as its features, and the values of the features are the probabilities obtained from the topic-document distribution. We compute classification accuracy using five-fold cross-validation and employ random forest, logistic regression, decision tree as the classification technique for both BBC news and 20 newsgroup datasets.

#### Baseline methods

This section adopts a data-driven approach. We employ meticulous comparative experiments to objectively assess the performance of our framework against existing methods for the news topic recognition. To rigorously evaluate the performance of the proposed method and examine the effect of diverse fusion approaches on the results, we will establish a comprehensive set of benchmarks. These include widely used techniques like TF-IDF, LDA, Word2vec, GloVe, SGL, CGL, SWL, and CWL, allowing us to directly compare our approach against established baselines and assess the added value of our fusion methods. The descriptions of the final four comparison models are as follows:

- SGL: Vector summation procedure on the text representations produced by the LDA and GloVe techniques.
- CGL: is performed by SGL. Combining the representation vectors acquired from the LDA and GloVe techniques.
- SWL: using the text representations produced by the word2vec and LDA methods.
- CWL: to perform a vector summing operation. combining the representation vectors produced by the LDA and word2vec techniques.

## Results and Discussion

This section, all experiments are performed on an Hp EliteBook with Intel Core i7-6200U, 2.30 GHz processor, 128 GB DDR4 RDIMM memory. In addition, the software environment is Python 3.8.5. Key libraries included Scikit-learn, Gensim and NLTK using Windows 10.

This study uses 20Newsgroup and BBC News dataset for classification. We follow a 70/30 split for training and testing, utilizing 70% of the data to train the model and 30% to evaluate its performance. On the BBC News dataset, our model flawlessly detects entertainment news with a perfect 100% accuracy. In the same way, the performance of the 20newsgroup dataset also improved. According to Du et al. (2022) the 20NewsGroup dataset poses a significant challenge, with the highest reported Micro-F1 value reaching only 0.83 due to factors like topic diversity and intra-topic similarity. Our approach in the 20NewsGroup slightly improved and reached at 0.88.

Table 5 shows the BBC news category identification results using the proposed WELDA approach.

**Table 5:** Results of BBC news dataset categories.

Categories	Precision	Recall	F1-Score	Suppport
Business	0.97	0.97	0.97	75
Entertainment	1.00	1.00	1.00	46
Politics	0.95	0.95	0.95	56
Sport	0.98	0.99	0.99	63
Tech	0.98	0.97	0.97	58

**Figure 3:**
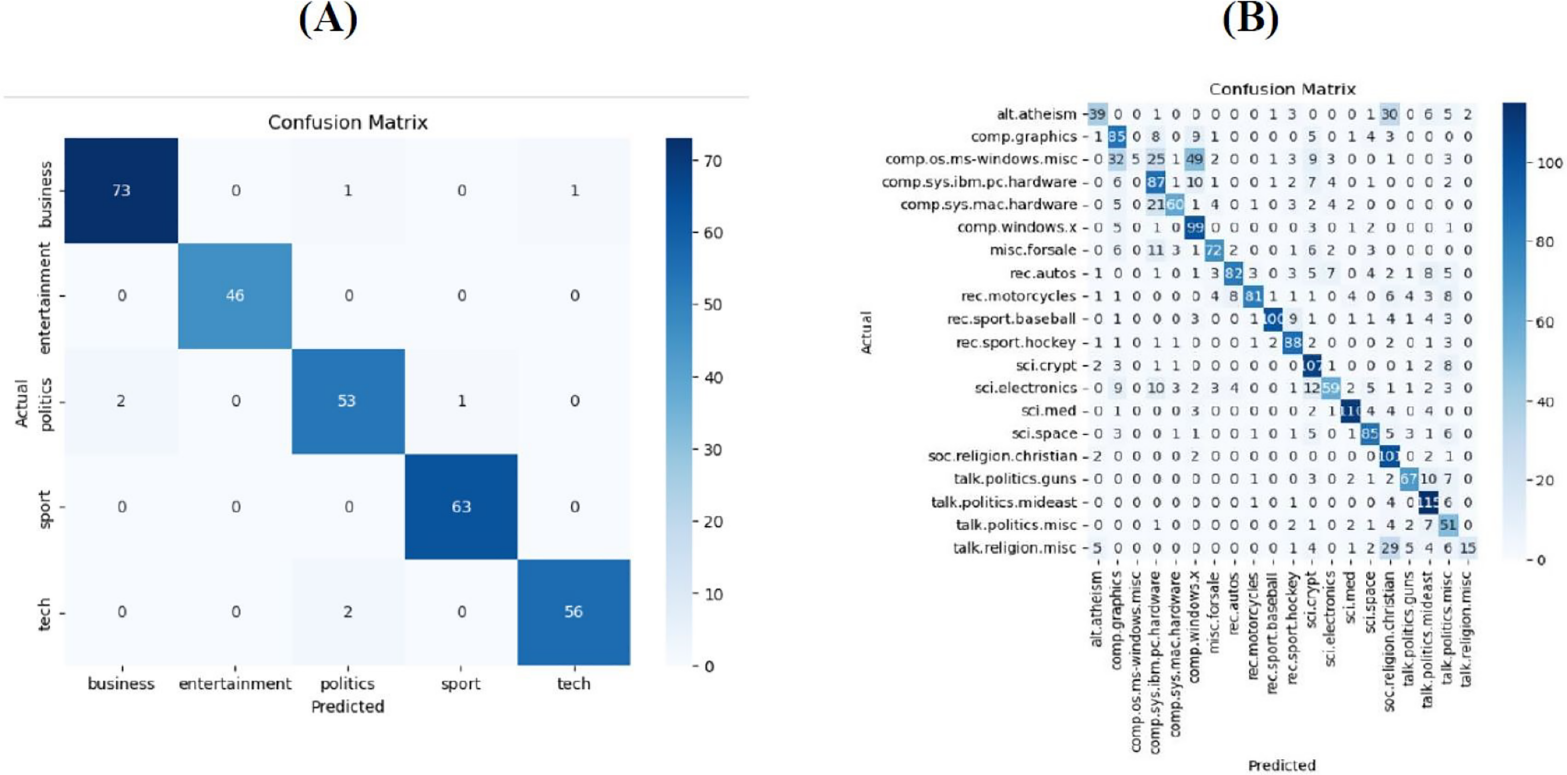
The confusion matrix reveals a high true positive rate indicating successful classification in this dominant case. (A) Depicts the confusion matrix on the BBC news dataset (B) presents the confusion matrix for the 20Newsgroups dataset.

Figure 3 illustrates the confusion matrix, revealing a high true positive rate, which indicates successful classification in this dominant case. Specifically, Fig. 3A depicts the confusion matrix for the BBC News dataset, while Fig. 3B presents the confusion matrix for the 20Newsgroups dataset. The matrices show that the model performs well on both datasets, with high True Positive rates in the majority of classes, indicating that the classification algorithm effectively distinguishes between different categories.

**Error analysis:** For the BBC dataset the model performs exceptionally well in classifying ‘business’ (73 correct) and ‘sport’ (63 correct) categories with minimal misclassifications. There are minor misclassifications in the ‘politics’ category, where two ‘politics’ documents are misclassified as ‘business’ and ‘tech’. This could be due to overlapping terminology related to political economy and technological policies. For 20 newsgroups dataset. The model performs well in most categories, with high correct classification rates in ‘comp.graphics’, ‘rec.sport.baseball’, ‘rec.sport.hockey’, and ‘sci.space’. There are significant misclassifications in categories such as ‘alt.atheism’, ‘talk.politics.guns’, ‘talk.politics.misc’, and ‘talk.religion.misc’. Certain categories like ‘sci.crypt’ and ‘sci.electronics’ have notable misclassifications. The confusion between ‘alt.atheism’ and other categories like ‘talk.politics.misc’ could be attributed to discussions on related topics that span multiple categories (*e.g*., religious discussions in political contexts). In both datasets, misclassifications occur in categories with semantically similar content. For instance, documents in ‘talk.politics.guns’ might frequently discuss legal aspects, which could overlap with ‘talk.politics.misc’.

**Figure 4:**
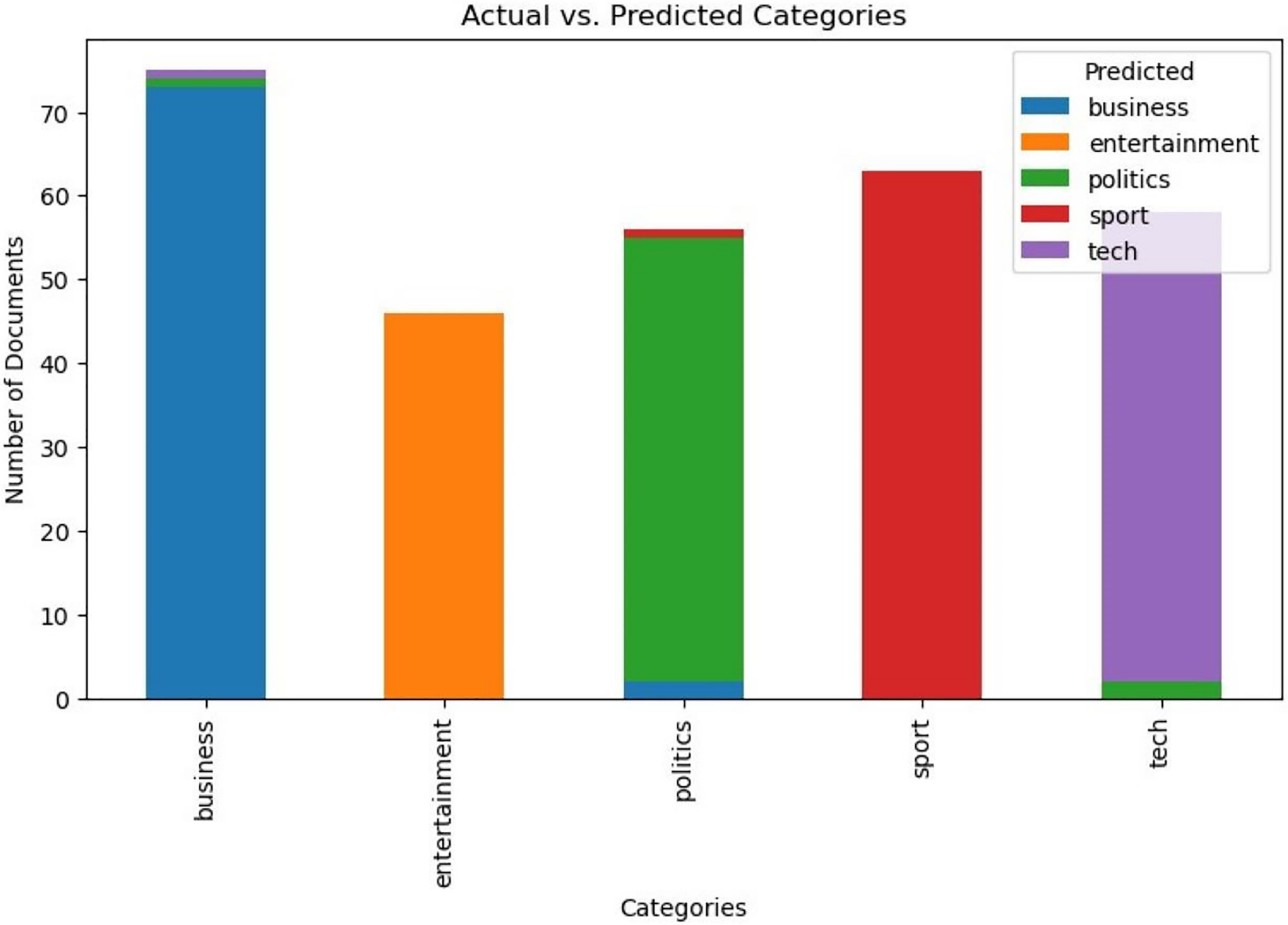
Bar chart of the actual *vs* predicted categories of BBC news dataset.

Figure 4 presents a bar chart comparing the actual *vs* predicted categories of the BBC news dataset. The bar chart illustrates the model’s performance in classifying the news articles into their respective categories, showing high agreement between the predicted and actual values. Based on the general observations, the model seems to perform extremely well on the BBC News dataset, with most instances being correctly classified. However, there are still some misclassifications happening in 20newsgroup dataset, particularly between categories that might share thematic similarities or overlapping topics.

Moreover, we apply classification algorithms for BBC news and 20NewsGroup dataset. We use logistic regression, random forest, and decision tree to compute classification accuracy.

**Figure 5:**
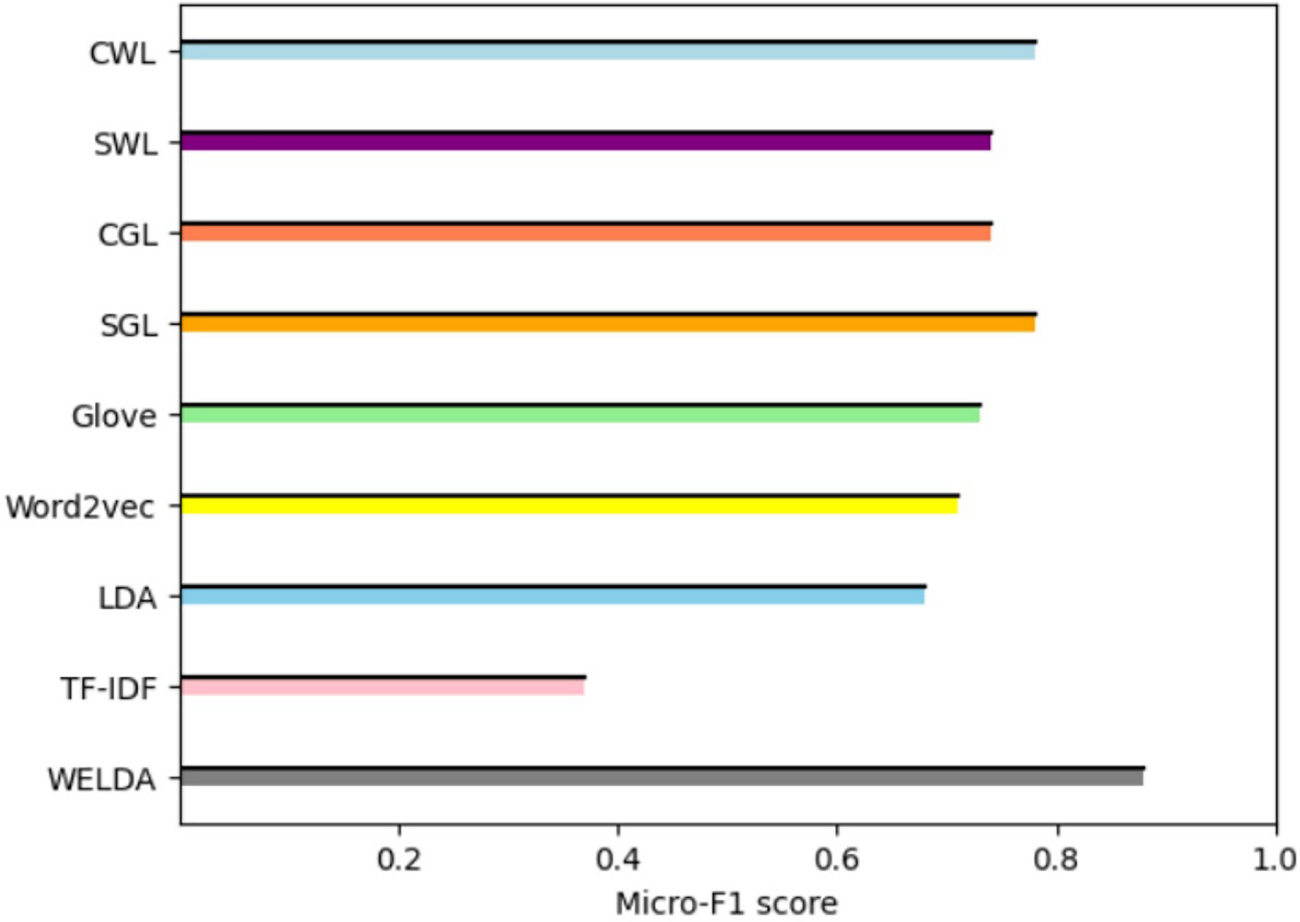
Comparison of WELDA with other baseline topic models.

Figure 5 presents the graphical visualization of the comparison of the results of 20NewsGroup dataset with different models. We concentrate on how vector dimensions affect the outcomes of the experiment. There is a need to mention that under the fusion technique, the vector dimension of the text representation based on the topic model and the word embedding model is the same. We also trained LDA models with correspondingly equal topic numbers. WELDA effectively handles polysemy and synonymy using word embeddings, while LDA struggles due to word co-occurrence patterns and TF-IDF’s raw frequency counts. Word2vec and GloVe embeddings excel but lack topic modeling. The value of Micro-F1 has a positive correlation with the final topic recognition effect. The proposed model WELDA uses rich word embeddings to improve text representation, which raises Micro F1 scores and improves discriminating. Its strong training methods improve its ability to generalize across the datasets.

Table 6 display Results of the 20NewsGroup in 20 classes for 7,532 texts by LR. According to the results of Khosa et al. (2023) the F1-score of different classification models like Logistic Regression, Random Forest and Decision Tree values are 0.79, 0.97 and 0.80. The results of RF are equal but their values of DT perform well.

**Table 6:** Results of classification accuracy.

	20Newsgroups	BBC News
Logistic regression	0.98	0.969
Random forest	0.979	0.978
Decision tree	0.50	0.815

For 20Newsgroups: All three models achieve high accuracy, exceeding 95%. Logistic Regression shows the best performance with 0.98 accuracy, followed closely by Random Forest (0.979) and then Decision Tree (0.50).

For BBC News: The pattern shifts slightly. While Logistic Regression remains in the lead with 0.969 accuracy, Random Forest comes very close with 0.978. Notably, Decision Tree performs considerably better here than on 20Newsgroups, reaching 0.815.

Logistic Regression seems to be the most consistently strong performer across both datasets, although Random Forest shows close results on BBC News. Decision Tree struggles on 20Newsgroups but improves significantly on BBC News, suggesting its suitability for specific data characteristics. 20Newsgroups appears to be a slightly easier dataset for classification due to the higher accuracy scores achieved by all models. BBC News presents a slightly more challenging task, particularly for Decision Tree.

For the baseline topic models the WELDA is compared to the other topic models TF-IDF, LDA, word2vec, GloVe, SGL, CGL, SWL, and CWL.

**Figure 6:**
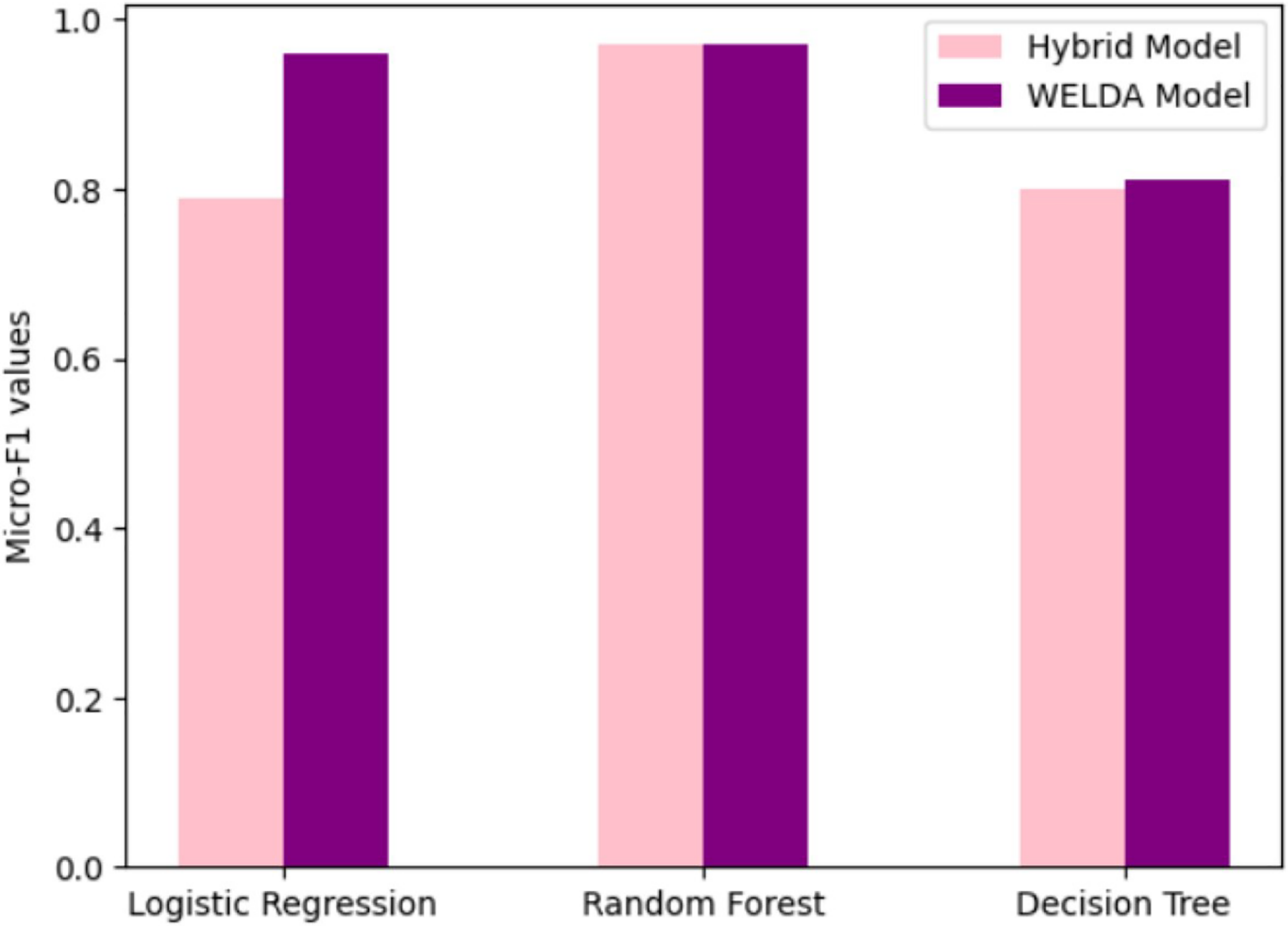
Comparison of classification values of WELDA with hybrid model of Khosa et al. (2023) on BBC news dataset.

Figure 6 presents the graph, highlighting the comparison of classification values. This figure compares the performance of WELDA with the hybrid model of Khosa et al. (2023) on the BBC News Dataset, demonstrating the differences and improvements in accuracy between the two models (Khosa et al., 2023).

The experimental results are shown in Table 7 taken from Du et al. (2022). Moreover, the model combination approach also pays attention to how vector dimensions affect the outcomes of the experiments. It is important to note that under the fusion technique, The topic model and the word embedding model-based text representation’s vector dimensions are equivalent. Trained word embedding models with 100 output vector dimensions for word2vec and GloVe. Likewise, for LDA models that have been trained and have an equal topic count. The value of Micro-F1 has a positive correlation with the final topic recognition effect. Our proposed model achieves better performance than other models.

**Table 7:** Results of the 20NewsGroup in 20 classes for 7,532 texts by LR.

Average 5-fold micro-F1 score	
Model	Logistic regression
TF-IDF	0.372
LDA	0.682
GloVe	0.710
SGL	0.732
CGL	0.782
Word2vec	0.740
SWL	0.747
CWL	0.780
WELDA	0.88

For validation of the model against real-world data we used dataset from Jalil et al. (2023) they collected news articles on three different topics. With the help of a web crawler, they crawled news articles linked to floods in Pakistan in 2022, the divorce news of Johnny Depp and Amber Heard, and the death of Queen Elizabeth II. For every topic, they gathered ten articles, with an average length of about 300 words. As a result, we got 30 thirty documents in all. We evaluated the topic coherence of our model using the C_v metric.

In Table 8, our model achieved an average coherence score of 0.617, with individual topic scores listed. The high topic coherence value shows that our model produces semantically meaningful and interpretable topics, validating its effectiveness for our application in topic modeling.

**Table 8:** Results of recent news articles.

Recent news article	Topic coherence
Topic 1	0.620867
Topic 2	0.539390
Topic 3	0.691178

To assess the scalability of our model, we used a dataset called “Recent News Articles” from a research study by Jalil et al. (2023). This dataset contains news articles on different topics collected from the web. This dataset was compiled by the authors by crawling web data on three diverse topics: celebrity news (divorce of Johnny Depp and Amber Heard), world news (death of Queen Elizabeth), and regional news (floods in Pakistan).

We performed experiments on both large benchmark datasets and small sized real-world data. The 20Newsgroups and BBC News datasets, as well as real-world data on the dataset of Recent News Articles. For the 20Newsgroups dataset (20,000 documents, 2 million words), the model achieved an average topic coherence score of 0.68, On the BBC News dataset (2,225 documents, 5.5 million words), it obtained an average score of 0.54. For the recent news data (30 documents, 300 words each on average), the average coherence score was 0.61, with a range of 0.53 to 0.69 as shown in Table 8. Our model is scalable, as evidenced by its constant performance on many datasets. The resilience and effectiveness of the model are demonstrated by its capacity to handle enormous datasets, such the 20Newsgroups and BBC News, which include millions of words, and still produce good coherence scores. Also, the model’s capacity to be successfully applied to smaller, real-world datasets confirms its versatility.

Furthermore, the Welda model, which is designed to work on various architectures, including microservices. This model ensures reliable performance with real-time data input since it is very flexible and can process data from several IoT devices as well as from a single source. Welda’s adaptability and effectiveness allow it to be used in a variety of deployment scenarios while preserving high levels of accuracy and consistency regardless of where the data comes from.

**Complexity analysis:** We evaluate the suggested WELDA method’s computational complexity against baseline models like LDA. The time complexity of LDA per iteration is *$O (K\cdot M\cdot l)$*, where K is the total number of topics, M is the total number of documents, and l is the average number of words per document. This implies that the algorithm must process every word in every page for every potential topic during each iteration.

The WELDA method, which integrates word embeddings with LDA, follows a similar pattern. There is an initial loading cost due to the pre-trained word embeddings, but this has no effect on the iterative complexity. The model’s complexity throughout each iteration can be written as *$O (K\cdot M\cdot (l + e))$*, where *e* is the additional computation due to word embedding. Since word embedding effectively capture semantic relationships, the additional component e remains smaller than l, ensuring that WELDA offers better semantic richness and topic coherence as comparable to typical LDA.

**Ablation study:** We performed an ablation analysis to understand the contribution of individual components in our WELDA model. Specifically, we examined how removing either the word embeddings or the topic modeling elements affected the model’s performance in terms of topic coherence and classification accuracy. We created two variants of the WELDA model:

- WELDA without word embeddings (LDA-Only):
- This variant uses the traditional LDA model without incorporating word embeddings. The goal is to evaluate the performance of topic modeling alone.
- WELDA without topic modeling (word2vec-Only):
- This variant uses word2vec embeddings to represent words and documents but does not apply the topic modeling component. This helps in understanding the impact of word embeddings alone.

In Table 9, the ablation studies clearly demonstrate that the word embeddings significantly enhance the model’s ability to represent semantic relationships, leading to improved topic coherence and classification accuracy. Meanwhile, the topic modeling component contributes to better generalization as LDA alone provides reasonable topic coherence. The word2vec component appears to play a crucial role in improving the model’s overall performance, particularly in terms of accurately classifying documents. The full WELDA model, which integrates both components achieve the 0.69 and 0.448 topic coherence respectively and 97% classification accuracy using random forest classifier in both datasets, which is the best performance across all metrics.

**Table 9:** Results of the ablation studies.

Dataset	Model	Topic coherence	Classification accuracy
20 Newsgroup	LDA	0.68025	44%
	Word2vec	0.67332	48%
BBC News	LDA	0.40191	74%
	Word2vec	0.43947	86%

**Statistical testing:** To validate the results observed in the WELDA model, we conducted a statistical test ANOVA, to compare the traditional LDA and word2vec model with proposed model ensure that the observed differences in topic coherence and classification accuracy are statistically significant. The result, with a p-value for classification accuracies is 0.0379 and for topic coherency is 0.0146. The values indicating that the improvements in topic coherence and classification accuracy of the WELDA model over the traditional approaches are statistically significant.

## Conclusions

As the big data era develops, we are dealing with real-world information overload issues that are getting worse. The aim for this research is to present a novel approach WELDA to combine the topic model with word embedding. We randomly resample words from this distribution during the Gibbs sampling inference. With this method, we utilize both the word co-occurrences across documents and the word-context co-occurrences at the same time. The topic model and word embedding model together can effectively leverage the topic distribution, semantic knowledge, and syntactic relationship to achieve better text representation through numerous experiments on the 20Newsgroup and BBC News datasets. We compare TF-IDF, LDA, word2vec, GloVe, DTE and WTE to proposed method, showing our approach is better for news topic recognition. The results convincingly demonstrate the superiority of our proposed method for news topic recognition.

Here are some areas worth investigating further:

1) The implementation of proposed method faced challenge during the computational complexity associated with integrating word embeddings and the need for extensive hyperparameter tuning. To address this issue, leveraging GPUs for parallel processing significantly reduced training times. Future researchers can use these findings as a roadmap for their particular applications.
2) The implication for media monitoring of the model’s accurately identifying and categorizing content, can assist in real-time analysis of news articles, social media posts, and other forms of digital media. WELDA’s ability to improve topic coherence makes it a robust tool for automated content analysis. Applications include summarizing large volumes of text data, detecting themes in customer feedback, and enhancing the capabilities of recommendation systems.
3) To further validate WELDA’s adaptability to real-world applications, future research should involves testing the model on data that is continuously updated, such as live news feeds or social media streams, to evaluate its real-time processing capabilities.

## Additional Information and Declarations

### Data Availability

The following information was supplied regarding data availability: The code is available at GitHub and Zenodo: - https://github.com/SidrahKaleem/Topic-Modeling-with-Embedding. - Sidrah Kaleem. (2024). SidrahKaleem/Topic-Modeling-with-Embedding: Word_embedding_empowerd_topic_model (Word_embedding_empowerd_topic_model). Zenodo. https://doi.org/10.5281/zenodo.12790060. The datasets are available at Kaggle and Scikit-learn: - https://www.kaggle.com/datasets/ervishvanathmetkari/bbc-news. - https://scikit-learn.org/0.19/datasets/twenty_newsgroups.html.
